# Experience and Reflection on the Treatment of Calcium Channel Blockers Poisoning: Two Case Reports of Elderly Patients

**DOI:** 10.1002/ccr3.71374

**Published:** 2025-11-20

**Authors:** Langhui Ou, Jintuan Lin, Haigang Zhang, Xiaoyi Xu, Siqi Lai

**Affiliations:** ^1^ Department of Critical Care Medicine Nanshan People's Hospital Shenzhen China

**Keywords:** calcium channel blockers poisoning, continuous renal replacement therapy, high‐dose insulin euglycemic therapy

## Abstract

Calcium channel blockers (CCBs) poisoning presents an extremely dangerous condition, with a particularly poor prognosis in elderly patients. Early identification, aggressive decontamination, and individualized hemodynamic and blood purification strategies significantly improve outcomes in elderly patients with CCBs poisoning.

**Summary**

- Optimizing outcomes in elderly patients with CCBs poisoning requires a bundle of key interventions: early identification, aggressive decontamination, high‐dose insulin euglycemic therapy (HIET), and individualized hemodynamic and blood purification support.
- Achieving this optimized management necessitates a multidisciplinary, patient‐centered approach that also addresses critical psychosocial elements.

## 1 Introduction

CCBs are commonly used cardiovascular drugs in clinical practice, primarily for the management of hypertension, angina pectoris and arrhythmia. However, excessive use of CCBs can lead to life‐threatening toxic reactions with a death rate ranging from 20% to 50% [1]. As the aging population increases, the number of CCB poisoning cases among elderly patients due to accidental ingestion or suicide is constantly increasing. Data released by America's Poison Center in 2022 indicated that cardiovascular drugs were the sixth leading cause of poisoning exposures. Among deaths related to poisoning, CCBs rank as the sixth most common class of drugs [2]. Elderly patients often suffer from multiple underlying diseases and have a lower tolerance to the toxicity of CCBs, which makes their treatment more challenging [3]. This article analyzed clinical data from two cases of elderly patients with CCB poisoning, aiming to explore the treatment strategies and clinical insights for CCB poisoning, thereby providing a reference for clinicians.

## 2 Case Report

### 2.1 Case 1

#### 2.1.1 Clinical Data

The patient was an 86 year‐old female who was admitted to the hospital on December 3, 2024, due to syncope lasting for 2.5 h. The patient had a history of hypertension for over 10 years, with the highest blood pressure reaching 180/100 mmHg. The patient has been taking amlodipine 5 mg once daily to control blood pressure. Approximately 2.5 h prior to admission, the patient experienced sudden syncope without any apparent precipitating factors, which resolved spontaneously after several seconds. Subsequently, the patient reported feelings of fatigue and did not seek medical attention promptly. The symptoms subsequently worsened, presenting as mottled skin on both lower extremities and cold extremities.

Physical examination on admission revealed: body temperature 36.5°C, heart rate 85 beats per minute, blood pressure 95/54 mmHg (maintained with dopamine at a rate of 8 μg/kg/min), and the patient was in a drowsy state. Coarse breath sounds were auscultated bilaterally in the lungs, accompanied by minimal crackles. The cardiac rhythm was regular without any murmur. Abdominal examination was unremarkable. Scattered mottling was noted on both lower extremities, and a slightly cool sensation was noted at the foot extremities.

Auxiliary examinations: Arterial blood gas analysis, complete blood count, and renal function test results are shown in the figure below. Cardiac marker tests showed an increase in troponin I from 0.093 ng/mL to 0.207 ng/mL and N‐terminal pro‐B‐type natriuretic peptide (NT‐proBNP) at 334 pg/mL. Coagulation function tests indicated D‐dimer levels at 2.18 μg/mL. The ionized calcium concentration decreased from 1.19 mmol/L to 0.39 mmol/L. Echocardiography indicated normal cardiac systolic function. Chest CT showed scattered infectious lesions in the dorsal segments of both lungs.

#### 2.1.2 Treatment Process

The primary diagnosis upon admission was unexplained shock. Despite the administration of dopamine and norepinephrine to maintain blood pressure, along with fluid resuscitation, and anti‐shock treatment, the patient's blood pressure remained difficult to maintain. Subsequently, additional medical history provided by the patient's family member indicated that the patient had accidentally ingested 48 tablets of amlodipine (a total of 240 mg), 17 h after the patient had taken the medication. Then, the patient was diagnosed with distributive shock caused by amlodipine poisoning and received treatment including plasma exchange, continuous renal replacement therapy (CRRT), continuous insulin infusion via micro‐infusion pump, and calcium supplementation.

During the patient's treatment, persistent anuria and elevated lactate levels (3.80 mmol/L) were observed; large doses of vasoactive drugs were required to maintain blood pressure. During plasma exchange treatment, the patient experienced a decline in oxygenation and undetectable blood pressure. Echocardiography and electrocardiogram showed electromechanical dissociation. Despite resuscitative efforts, the patient could not be revived.

### 2.2 Case 2

#### 2.2.1 Clinical Data

The patient was a 77‐year‐old female, who was admitted to the hospital on February 19, 2025, due to recurrent cough, sputum production, and dyspnea over 7 years, with symptoms worsening and leading to loss of consciousness over the past 2 h. The patient has a history of chronic obstructive pulmonary disease (COPD), stage 5 chronic kidney disease (CKD) (undergoing maintenance hemodialysis), percutaneous coronary intervention (PCI) for coronary heart disease, and grade 3 hypertension (extremely high risk). Two hours prior to admission, family members noted her loss of consciousness. The pulse oximetry measured at home was 80%, and the blood pressure was at 79/43 mmHg.

Physical examination upon admission: the patient was in a state of shallow coma, with a body temperature of 36.5°C, a heart rate of 75 beats per minute, and a blood pressure of 94/64 mmHg (maintained by dopamine at a rate of 5 μg/kg/min). Respiratory distress was observed, with wheezing audible in both lungs. The cardiac rhythm was regular without any pathological murmurs. Abdomen distended and cold extremities were noted.

Auxiliary examinations: Arterial blood gas analysis, complete blood count, and renal function test results are shown in the figure below. Bedside echocardiography showed decreased myocardial contractility. Chest CT showed infection in the lower lobe of the right lung.

The differences in laboratory test results of the two cases are shown in Tables 1, 2, 3.

**TABLE 1:** Arterial blood gas analysis report.

Test	Value		Unit	Reference range
	Patient 1	Patient 2		
pH	7.263	7.062 ↓	—	7.35–7.45
PCO_2_	42.3	114.60 ↑	mm Hg	35–45
PO_2_	101	182 ↑	mm Hg	80–105
BE	−6	2	mm Hg	−2‐3
HCO_3_^−^	19.1	32.6 ↑	mm Hg	22–26
Lactate	2.40 ↑	5.3 ↑	mmol/L	< 2
O_2_ concentration	40	29	%	

**TABLE 2:** Complete blood count report.

Test	Value		Unit	Reference range
	Patient 1	Patient 2		
WBC	12.7 ↑	6.6	× 10 ^ 9/L	3.5–9.5
Hb	117 ↓	101 ↓	g/L	115–150
PLT	296	125	× 10 ^ 9/L	125–350
CRP	< 0.2	0.66	mg/L	< 5

Abbreviations: CRP, C‐reactive protein; Hb, hemoglobin; PLT, platelet count; WBC, white blood cells.

**TABLE 3:** Renal function test report.

Test	Value		Unit	Reference range
	Patient 1	Patient 2		
BUN	7.3 ↑	18.1 ↑	mmol/L	3.2–7.1
CRE	97.1 ↑	468.5 ↑	μmol/L	46–92

Abbreviations: BUN: blood urea nitrogen; CRE: creatinine.

#### 2.2.2 Treatment Process

**FIGURE 1:**
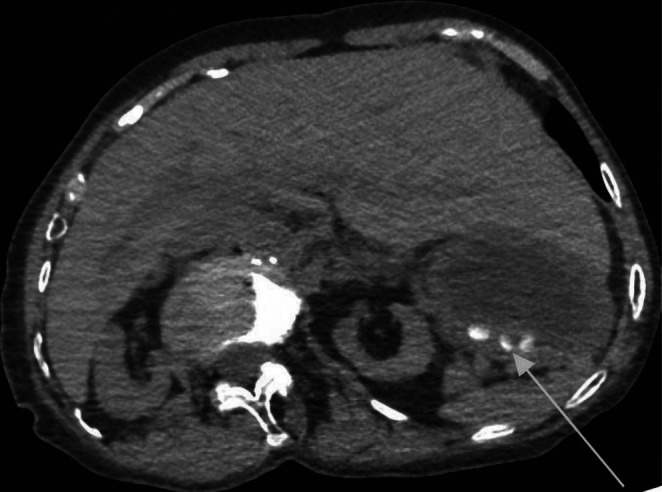
Axial CT scan of the abdomen in Case 2 showing multiple hyperdense structures in the stomach (arrow), consistent with retained drug fragments. The finding supported the decision to perform catharsis.

The primary diagnosis was acute exacerbation of COPD, Type II respiratory failure, stage 5 CKD. Upon admission, the patient underwent CRRT, noninvasive ventilation, and maintenance of blood pressure with vasoactive drugs. Further inquiry into the medical history revealed that the patient has ingested eszolam (50 tablets), sacubitril/valsartan (30 tablets), and nifedipine sustained‐release tablets (approximately 1800 mg). It has been 5 h since the patient took the above medication. Abdomen CT on admission showed drug fragments in the stomach (Figure 1). Subsequently, blood samples were sent for therapeutic drug monitoring. The results indicated that the concentrations of nifedipine (385 ng/mL) and eszolam (550 ng/mL) were both abnormally elevated, suggesting drug poisoning. Therefore, the treatment plan was adjusted as follows: (1) blood purification: CRRT combined with hemoperfusion for drug clearance; (2) high‐dose insulin combined with glucose therapy: insulin at 2 U/kg/h to maintain blood glucose level between 8 mmol/L and 10 mmol/L; (3) calcium supplementation: ionized calcium level was maintained within the range of 1.77–2 mmol/L; (4) laxative: polyethylene glycol combined with sodium phosphate powder was administered as a laxative until a large number of tablets could be observed to be excreted; (5) circulatory support: adequate fluid resuscitation was provided, and norepinephrine combined with dopamine was used to maintain blood pressure.

On the third day of treatment, the patient regained consciousness, shock was corrected, and the use of vasoactive drugs was discontinued. During hospitalization, complications such as abdominal pain and abnormal coagulation function occurred. However, these were improved through multidisciplinary collaboration. The patient was eventually discharged in good condition.

## 3 Discussion

### 3.1 Prevalence of CCBs Poisoning

CCBs poisoning accounts for a relatively small proportion of drug poisonings globally (typically < 1%) [2], but it is one of the leading causes of death from cardiovascular drug overdose and carries a high clinical severity. The absolute number of cases has stabilized with increasing prescription volume, and CCBs poisoning is more common in regions such as North America and Europe. Despite being relatively rare, CCBs poisoning has a mortality rate as high as 2%–5%, and can exceed 20% if cardiogenic shock occurs [3], making it a disproportionately high‐risk group among all poisonings.

### 3.2 Pathophysiological Mechanism of CCBs Poisoning

CCBs inhibit the influx of calcium ions by blocking L‐type calcium channels, thereby reducing the contractility of myocardial cells and the tone of vascular smooth muscle [4]. Overdose of CCBs may lead to: (1) Cardiovascular system: severe hypotension, bradycardia, conduction block, cardiogenic shock. (2) Metabolic disturbances: acidosis, hyperlactatemia, hypocalcemia. (3) Other systems: central nervous system depression, intestinal obstruction, noncardiogenic pulmonary edema. Case 1 presented with typical refractory hypotension, hypocalcemia, and metabolic acidosis. In addition to circulatory failure, case 2 was also complicated by COPD and CKD.

### 3.3 Comparison of Treatment Strategies and Analysis of Effects

The differences in treatment strategies and outcomes of the two cases are shown in Table 4:

**TABLE 4:** Comparison of treatment strategies and outcomes for two cases of CCBs poisoning.

Therapeutic elements	Case1 (amlodipine poisoning)	Case2 (nifedipine poisoning)
Early identification	Delay (Family members provide medication history only after admission)	Relatively timely (detecting gastric drugs through CT, early completion of drug blood concentration tests)
Gastrointestinal decontamination	Not implemented early	Early active catharsis (expulsion of large pills)
Calcium supplements	Regular doses	High dose (maintenance ionic calcium 1.77–2 mmol/l)
Insulin therapy	Conventional micropump	High dose (2 U/kg/h) combined with glucose
Blood purification	CRRT combined with plasma exchange	CRRT combined with hemoperfusion
Multidisciplinary assistance	Limited	Multidisciplinary consultation including critical care, nephrology, psychiatry, et.

The possible reasons for the treatment failure in case 1 included the following: first, the patient administered an extremely high dosage of amlodipine (240 mg), and its high protein binding rate (93%–98%) limits the effectiveness of blood purification. Second, the patient did not seek medical attention in a timely manner, thus delaying diagnosis. Given that patient 1 is elderly with poor organ reserve function, this further diminished therapeutic efficacy. In contrast, in case 2, the condition was identified early and medical care was promptly sought. During treatment, a thorough catharsis was performed to effectively eliminate residual drugs from the body. Standardized application of hemoperfusion integrated extracorporeal treatment individualized blood purification strategies and multidisciplinary collaborative management also played important roles.

### 3.4 Critical Measures for High‐Risk CCBs Poisoning

Based on the experience from these cases and the existing literature, we summarize the critical therapeutic measures for high‐risk CCBs poisoning as follows:

1. Early identification and diagnosis: detailed inquiry about medication history (type, dosage, and timing). Be vigilant of atypical manifestations. Case 2 initially presented with an acute exacerbation of COPD as the main symptom. Comprehensive early laboratory tests should be conducted, including blood gas, lactate, electrolytes (especially calcium ions), and drug concentration testing.
2. Gastrointestinal decontamination: gastric lavage is most effective within 1 h after ingestion. Based on the successful experience in case 2, gastric lavage can be performed using activated charcoal with an initial dose of 1 g/kg followed by 0.5 g/kg every 4 h. Catharsis can be achieved using sodium phosphate or polyethylene glycol electrolyte solution until clear stool is discharged. Previous cases have confirmed that this is a key treatment method [5, 6]. It is particularly emphasized that for exposure to sustained‐release formulations where poisoning is anticipated, if blood pressure remains normal and intestinal perfusion is adequate, consider total bowel irrigation using high‐molecular‐weight polyethylene glycol solution [7, 8].
3. Circulatory support: fluid resuscitation uses crystalloid at a rate of 20 mL/kg while avoiding excessive administration. Vasoactive drugs are primarily norepinephrine (0.1–1 μg/kg/min). Calcium administration involves calcium chloride (10% 10–20 mL) or calcium gluconate to maintain free ionized calcium > 1.5 mmol/L. [5, 6].
4. HIET: High‐dose insulin (HDI) exerts its effects through the following four mechanisms [9]: (1) increasing cardiac output in myocardial cells via calcium‐dependent and calcium‐independent pathways. This increase in cardiac output mainly results from an increased stroke volume rather than heart rate. (2) Optimizes myocardial energy utilization by saturating myocardial insulin receptors, which enhances glucose availability for ATP production. (3) HDI can improve endocrine dysfunction caused by CCBs poisoning leading to hyperglycemia. (4) Enhancing endothelial nitric oxide synthase to produce vasodilation effects, which increases cardiac output and improves microvascular dysfunction associated with cardiogenic shock. HDI may be used to treat cardiogenic shock caused by severe CCBs poisoning [10]. Specific usage includes: (1) administer insulin at 1 U/kg intravenously as a loading dose. Maintain insulin at 0.5–2 U/kg/h up to a maximum of 2–10 U/kg/h while combining with 25% or 10% glucose at a rate of 0.5 g/kg/h. Monitoring blood glucose levels hourly and potassium levels is monitored every 2 h.
5. Blood purification techniques: CCBs are considered nondialyzable drugs with clearance rates exceeding 400 mL/min. Current pharmacokinetic and toxicokinetic data indicate that extracorporeal clearance can increase total clearance by 5% to 10% at most [11]. Although the Extracorporeal Treatment Working Group does not recommend the extracorporeal methods to enhance the clearance of amlodipine, diltiazem, and verapamil in severe poisoning situations [11], in certain specific situations such as severe hypotension, shock, peri‐cardiac arrest, or even cardiac arrest, where tissue perfusion is severely compromised and there is severe liver and kidney dysfunction, endogenous clearance capacity will be significantly affected. In such cases, blood purification may become the primary or even the only means of clearance [6]. CRRT is suitable for restoring acid–base and electrolyte homeostasis, particularly in cases of acute kidney injury. Plasma exchange is appropriate for CCBs with high protein binding rates (such as amlodipine). Hemoperfusion mainly removes lipophilic drugs (successful application in case 2).
6. Other supportive treatments: a 20% lipid emulsion (Intralipid) at an initial dose of 1.5 mL/kg intravenously, followed by a continuous infusion at a rate of 0.25 mL/kg/min for 30–60 min. However, a large retrospective study found no benefit from the use of lipid emulsion in CCBs poisoning, and both experimental and clinical data suggest that lipid emulsion may increase the gastrointestinal absorption of lipophilic drugs [12]. Temporary pacing is recommended for patients with severe bradycardia unresponsive to atropine.

### 3.5 Specificities of CCBs Poisoning in the Elderly

CCBs poisoning is more severe among elderly patients, and the mortality rate is significantly higher than that in the younger population. A retrospective analysis based on 52 cases of CCBs poisoning showed that age is an independent risk factor affecting patients' prognosis. The older the patient, the higher the mortality rate [13]. Specifically, CCBs poisoning in the elderly also has its particularities. Elderly patients are prone to accidental ingestion (case 1) or coexisting mental disorders (case 2). They often present atypical neurological symptoms such as common drowsiness, delirium, or coma (case 2), which may be misdiagnosed as cerebrovascular disease or an aggravation of pre‐existing underlying diseases. Elderly patients often have multiple underlying diseases and reduced tolerance, particularly concerning cardiovascular system damage. For instance, they may experience refractory hypotension and cardiogenic shock due to their relatively poor baseline cardiac function combined with peripheral vasodilation and myocardial contractility inhibition, along with their poor response to vasopressor drugs. The decreased hepatic and renal function in elderly patients leads to delayed drug clearance and prolonged poisoning time. Complications are accompanied by liver and kidney function impairment and severe metabolic acidosis. Moreover, the decline in liver and kidney functions makes it more difficult to correct acidosis in these patients. Both cases presented varying degrees of metabolic acidosis. Case 1's death was closely related to the advanced age of 86 years and poor multiorgan functional reserve.

### 3.6 Clinical Reflection

The main challenge encountered during the treatment of case 1was the refractory shock that was hard to correct. The extremely high protein binding rate of amlodipine limited the effect of blood purification. Additionally, the compensatory capacity of the cardiovascular system in the elderly patients is generally diminished, and delayed medical treatment can lead to irreversible organ damage. All these factors may have contributed to the outcome. Eventually, the patient succumbed despite maximal supportive measures.

The successful experience from case 2 indicates that early identification is foundational to treatment, and an integrated therapeutic strategy is essential. Furthermore, psychological and mental intervention should not be overlooked (the patient exhibited a clear suicidal tendency). Prior to discharge, a psychiatric consultation was provided to prevent any potential recurrence of suicide attempts.

### 3.7 Psychiatric Evaluation

In instances of CCBs overdose (as illustrated in Case 2), a psychiatric evaluation plays a crucial role in the overall treatment approach [14]. The primary objectives of this assessment include: understanding the intent behind the overdose, whether it be suicidal, accidental, or related to nonsuicidal self‐injury, which serves as the central focus of the evaluation; identifying any concurrent mental health conditions (such as major depressive disorder, bipolar disorder, or anxiety disorders), which are significant risk factors for self‐harming behaviors; and guiding further clinical management. This involves delivering immediate crisis intervention for acute cases, developing safety strategies, and arranging follow‐up psychiatric care and long‐term therapeutic plans to support sustained recovery.

## 4 Conclusion

CCBs poisoning presents an extremely dangerous condition, with a particularly poor prognosis in elderly patients. Early identification and intervention are necessary.

The comprehensive treatment strategy includes gastrointestinal decontamination, circulatory support, HIET and blood purification, etc.

Case 1 Death was related to extremely high‐dose amlodipine poisoning and delayed medical treatment; Case 2: Recovery was attributed to early identification and standardized treatment.

The treatment of CCBs poisoning in the elderly requires individualized plans and multidisciplinary collaboration, while also paying attention to psychological and social factors.

## Author Contributions

**Langhui Ou:** data curation, formal analysis, investigation, project administration, supervision, writing – original draft, writing – review and editing. **Jintuan Lin:** supervision, writing – review and editing. **Haigang Zhang:** supervision, writing – review and editing. **Xiaoyi Xu:** writing – original draft. **Siqi Lai:** writing – original draft.

## Ethics Statement

Patient personal data have been respected per institutional guidelines.

## Consent

Written informed consent was obtained from the patient in Case 2 and the legal guardian of the deceased in Case 1 for publication.

## Conflicts of Interest

The authors declare no conflicts of interest.

## Data Availability

All data generated or analyzed in this study are included in this published article. Additional inquiries can be addressed to the corresponding author.
